# Synthesis of Tantalum Carbide Using Purified Hexane by Titanium Powder

**DOI:** 10.3390/ma15217510

**Published:** 2022-10-26

**Authors:** Seon-Min Hwang, Ji-Won Hong, Yong-Ho Park, Dong-Won Lee

**Affiliations:** 1 Department of Materials Science and Engineering, Pusan National University, 2 Busandaehak-ro 63 Beon-gil, Busan 46241, Korea; 2 Titanium Department, Korea Institute of Materials Science (KIMS), 797 Changwon-daero, Changwon 51508, Korea

**Keywords:** purification of hexane, gaseous carburization, tantalum carbide, commercial hexane, Ti powder, metal oxidation

## Abstract

Hexane is a safe, efficient, and cost-effective alternative to other commercial hydrocarbons for gaseous carburization; however, commercial hexane is not sufficiently pure. Titanium powder can remove oxygen-containing impurities from commercial hexane; however, research on the use of titanium powder remains limited. We investigated the purification of hexane using titanium, copper, and aluminum powders and used the purified hexane for the gaseous carburization of tantalum. Ti exhibited lower activation energy for oxidation (1.55 kJ/mol) than Cu (91.09 kJ/mol) and Al (150.25 kJ/mol) and a significantly higher oxidation rate (0.0269 g/h) in hexane at room temperature than Cu (0.0018 g/h) and Al (0.0001 g/h). The carbon content in tantalum carburized using the purified hexane was comparable to that carburized using unpurified hexane (approximately 6.22%); however, its oxygen content was significantly lower (1.39%), which indicates that the produced tantalum carbide has a higher purity. X-ray diffraction results revealed that the oxidation products of tantalum, such as Ta_2_O, TaO_2_, Ta_0.8_O_2_, and Ta_2_O_5_, were absent in the sample carburized using the purified hexane. Therefore, Ti powder can effectively remove oxygen-containing impurities from commercial hexane and facilitate its use as an effective carburizing medium for the synthesis of high-purity tantalum carbide.

## 1. Introduction

Owing to their unique properties, such as high corrosion resistance, excellent high-temperature mechanical properties, thermal and chemical resistance, low thermal expansion, wear resistance, and high hardness, carbide-based materials are receiving considerable attention [1]. These materials are used in various fields, such as automotive, aerospace, nuclear, and electronics, as well as in various industrial abrasives, cutting, and grinding tools [2]. Among these materials, tantalum carbide is known for its high melting point and hardness. It is synthesized partly via a sintering process, using powders as raw materials, and is applied for the manufacturing of various products such as cutting tools and dies [3,4,5,6]. In particular, tantalum carbide has a high utility value as an additive for improving the performance of materials for carbide tools, such as tungsten carbide, because its presence in small amounts improves wear resistance and machinability [7,8,9,10].

Tantalum carbide powders are mainly manufactured via three methods: (1) heat treatment in a non-oxidizing atmosphere using a mixture of tantalum metal and graphite powders, (2) solid carburization, such as self-propagating high-temperature synthesis (SHS) using the reaction heat between reactants by mixing tantalum and graphite powders [11,12,13], and (3) gaseous carburization using hydrocarbon gases such as methane, propane, and butane as the carburizing media for tantalum [14,15]. In addition, other methods such as reduction carburization, which employs a mixture of tantalum oxide (Ta_2_O_5_) and graphite powders to simultaneously induce reduction and carburization reactions, are being studied for commercialization [16].

Among these methods, solid carburization is limited by requirements of high reaction temperatures of 2000 K or higher, difficulties in controlling product uniformity, and residual-free carbons [17]. Furthermore, solid carburization can be hazardous due to the CO gas generated during the reaction [16]. Meanwhile, gaseous carburization requires the use of highly pure hydrocarbons to prevent the re-oxidation of tantalum carbide and ensure effective carburization. Commercial propane and butane are unsuitable for carburization because of their low purity, and the cost increases because many processing devices for purification are required to use these [18,19]. Although high-purity methane is commercially available and is used for carburization, it is expensive and economically unfeasible [20]. Therefore, this study aims to synthesize tantalum carbide using a hydrocarbon gas produced from liquid hexane, which is less expensive and easier to handle than the conventional methane used for carburization.

The purity of hexane commonly used in the industry is 95%, necessitating a process for the removal of impurities. However, there have been no reports on the removal of impurities from commercial hexane. For the first time, we have suggested the economically feasible method for producing of TaC ceramic compound material. We purified the commercially available hexane using irregularly shaped titanium powder, which is further used to produce the high purity TaC by means of gas carburization [21], as it can capture most of the oxygen-containing compounds that constitute a majority of the impurities in hexane. Therefore, the present study investigated the purification of hexane using not only titanium powder, but also other universal and recyclable metal powders such as iron, copper, and aluminum. However, the study employed magnets, to which the iron powder stuck during stirring, making it difficult to evaluate the reliability of the impurity-removal reaction. Therefore, we attempted to systematically verify the purification of hexane using titanium, copper, and aluminum powders by synthesizing tantalum carbide using a hydrocarbon gas obtained from purified hexane. Titanium was found to be more effective at the removal of oxygen-containing impurities from hexane than copper and aluminum at room temperature, and hexane purified using titanium powder was proven to yield high-purity tantalum carbide when used as a carburizing agent for tantalum.

After all, in this study, in order to manufacture high-purity tantalum carbide more easily and efficiently compared to the conventional carburizing process, we intended to produce high-purity tantalum carbide more safely and with less energy and cost.

## 2. Materials and Methods

Hexane with 95% purity was used (Samchun Chemical Co., Ltd., No. 000H0113, Seoul, Korea). The metal powders to be used for hexane purification—Ti, Cu, and Al powders—were obtained (Changsung Ltd., Incheon, Korea), with purities of 99.99% and average particle sizes of 30–40 μm. Furthermore, in a recent study, we manufactured tantalum metal powder using tantalum oxide (Ta_2_O_5_) as the raw material with reducing magnesium gas. Therefore, the tantalum powder for the carburizing reaction was prepared as per the method reported in our previous study [22].

In a branched Erlenmeyer flask, 500 and 10 g of hexane and metal powder, respectively, were placed, and the inlet was closed with a stopper. The mixture was then stirred using a magnetic stirring bar. The temperature was increased at a rate of 5 K per minute to the preset stirring temperatures of 293, 308, and 323 K and then maintained at respective temperatures for 3 h for the purification of hexane.

**Figure 1:**
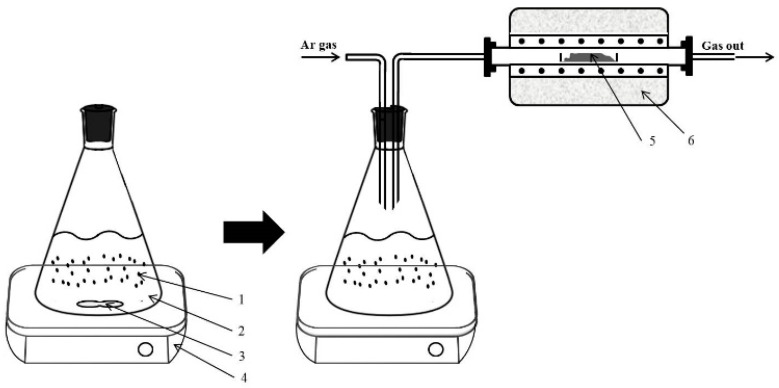
Synthesis of TaC powder using the purified hexane gas. Here, 1–6 indicate the metal powder, hexane, magnetic stirrer, stirring hot plate, reduced tantalum powder, and tube furnace, respectively.

**Figure 2:**
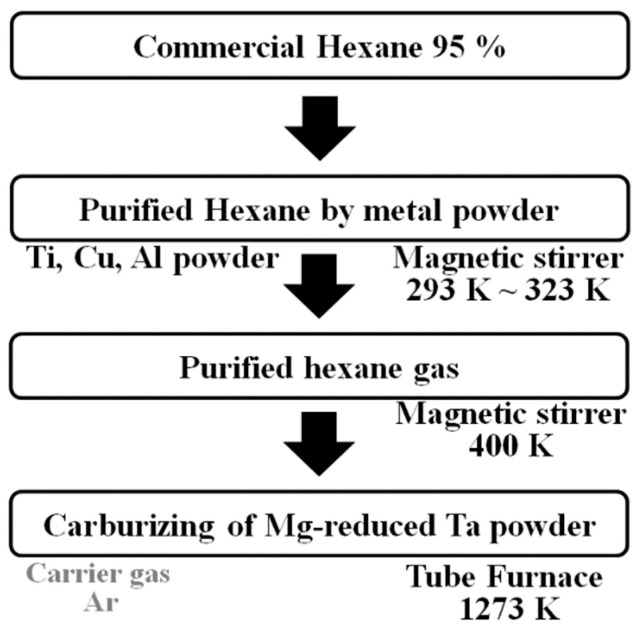
Flow chart of synthesis of TaC powder using the purified hexane gas.

After the purification reaction, the temperature of the flask was increased to 400 K using a heating plate, which is higher than the boiling temperature of hexane (342 K). The tantalum metal powder was heated to 1273 K using the tube furnace at a heating rate of 10 K/min. The flask was connected to the tube furnace using a stainless-steel tube. Argon gas (99.999% pure) was selected as the carrier gas, which flowed through the branch of the flask at a flow rate of 200 cc/min, such that the hydrocarbon gas could reach the tube well. The hydrocarbon gas supplied to the tube through the argon gas reacted with the tantalum metal powder for 2 h at 1273 K. The temperature was raised at 10 K per minute up to the reaction temperature. The tantalum carbide powder was obtained by cooling the carburization reaction mass. Figure 1 and Figure 2 shows the set-up and work flow for the purification process of hexane and carburization of the tantalum powder.

The metal powders used for the purification of hexane were subjected to specific surface area and morphological analyzes, such as the Brunauer–Emmett–Teller (BET) analysis (Specific Surface Area Analyzer, 3Flex, Micromeritics Instrument Corporation, Norcross, Georgia, USA) and scanning electron microscopy (SEM, MIRA3-LM, TESCAN, Brno, Czech Republic). The tantalum carbide powder obtained via carburization was subjected to characteristic evaluation of phases and components using an X-ray diffractometer (D/Max-2500, Rigaku International Corporation, Tokyo, Japan), an oxygen/nitrogen/hydrogen determinator (ONH-P, ELTRA, Haan, Germany), and a carbon/sulfur determinator (CS-200/LECO, LECO, St. Joseph, MI, USA).

## 3. Results and Discussion

**Figure 3:**
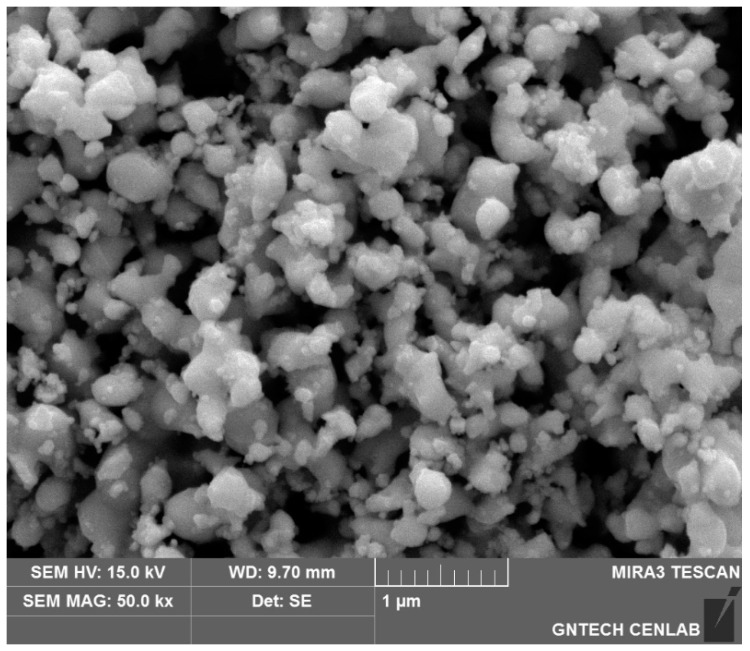
Scanning electron microscopy (SEM) image of Mg-reduced tantalum powder.

**Figure 4:**
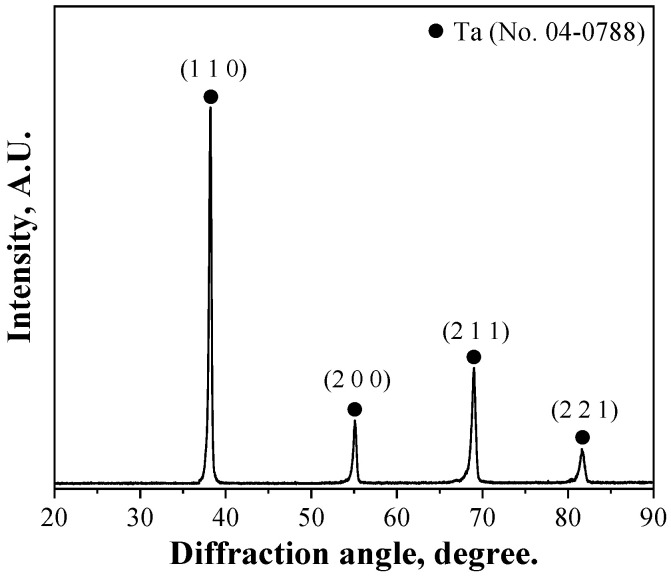
X-ray diffraction (XRD) patterns of the Mg-reduced tantalum powder.

Figure 3 and Figure 4 show the SEM and X-ray diffraction (XRD) results of the previous study, respectively [22]. After controlling the particle size of tantalum oxide (Ta_2_O_5_) via heat treatment at 1573 K for 2 h, magnesium reduction was performed at 1173 K in an argon atmosphere for 20 h to prepare a Ta + MgO mixture. SEM and XRD analyzes were then performed on the pure tantalum powder, which was obtained by removing magnesium oxide, a byproduct of the reduction, by stirring and washing the reaction mass with an aqueous hydrochloric acid solution. SEM shows that the particles of the reduced tantalum metal powder are spherical, with an average size of 200–250 nm. The reduction was considered successful, based on the absence of magnesium oxide from the tantalum phase, as indicated by the XRD results of the reduced tantalum powder (see Figure 4). The oxygen and inductively coupled plasma (ICP) analyses of the prepared tantalum powder indicated oxygen and magnesium contents of 0.36 and 0.005 wt.%, respectively [22].

**Figure 5:**
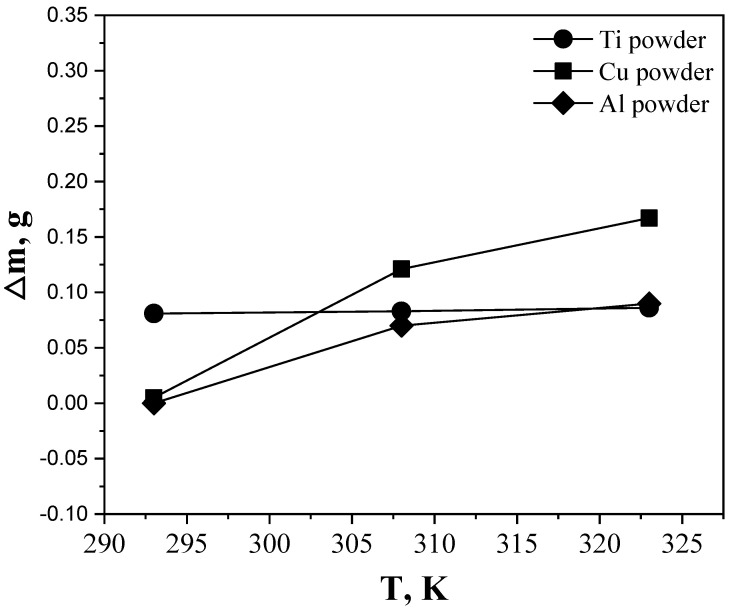
Changes in the weight of the metal powders used for the purification of hexane at various stirring temperatures.

Figure 5 shows the variation of the weights of Ti, Cu, and Al powders used for the purification of hexane with the stirring temperature. The increase in the weights of the metal powders after stirring was attributed to their combination with the oxygen present in hexane as an impurity. Unlike the other two powders (Al and Cu), the Ti powder underwent a weight change of approximately +0.081 g at 293 K. The changes in the weights of Ti, Cu, and Al powders were +0.083, +0.121, and +0.065 g, respectively, at 308 K, and +0.086, +0.167, and +0.086 g, respectively, at 323 K.

The oxygen-containing compounds present as impurities in hexane may include dissolved oxygen (O_2_), moisture (H_2_O), sulfuric acid (H_2_SO_4_), and carbon dioxide (CO_2_) [21]. G. Graziano [23] reported that because of the weak dispersion attraction, the solubility of oxygen is poor in hexane. Reportedly, 100 g of commercial hexane contains approximately 0.08 g of dissolved oxygen [24]. However, the results shown in Figure 5 indicate that the weight change of Ti powder stirred at 293 K was 0.081 g, greater than the contents of dissolved oxygen. This weight change was attributed to the reaction with oxygen contained in sulfuric acid and carbon dioxide in addition to that with the dissolved oxygen and moisture present in the hexane.

Moreover, as shown in Figure 5, the weight change trends differed with the type of metal powder, owing to the differences in the oxidation rates of the metals. The increased weight relative to the initial weight corresponded to the amount of metal oxidized, and by dividing the difference by the stirring time, the oxidation rate, k, was obtained. Table 1 summarizes the calculated oxidation rates of the metals. It is noticed that at room temperature (293 K), the oxidation rate (k) of Ti is much higher than that of Cu and Al.

Moreover, the oxidation rates of all metals increased with the temperature of hexane solution, depending on their activation energies. The activation energy for the oxidation reaction of each metal was determined from the slope of the line obtained from the relationship between ln k and the reciprocal of the hexane solution temperature (1/K) [22].

**Figure 6:**
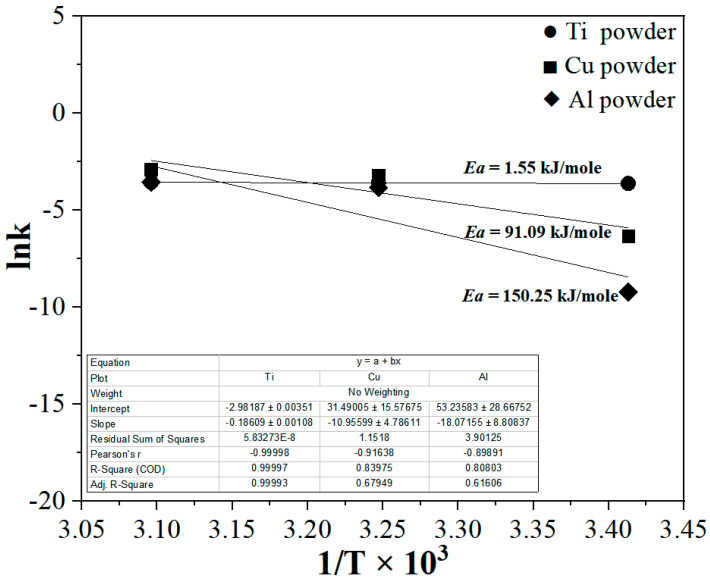
Plot of ln k versus 1/T × 10^3^ for the estimation of the activation energies of the metal powders used for the purification of hexane at various stirring temperatures.

Figure 6 shows the calculated activation energies for oxidation behaviors of the metals. The activation energies of Ti, Cu, and Al powders were 1.55, 91.09, and 150.25 kJ/mole, respectively, which indicates the rate of oxidation is much faster with Ti.

The oxidation of metals generally occurs at temperatures more than half of their melting point, and not at temperatures as low as room temperature. However, oxidation was observed even at room temperature in this study and was attributed to a behavior different from the general oxidation behavior.

The oxidation of the metals in hexane at room temperature was attributed to the corrosion of the metals in the fluid [25]. The basic principle of corrosion is as follows: the exposure of a metal to an environment containing water and oxygen results in (1) an oxidation reaction, in which the metal generates electrons and is converted to metal ions and (2) the reduction of oxygen by the electrons generated from the metal. Subsequently, metal hydroxides are formed by the reaction of the hydroxide and metal ions formed by the oxidation and reduction reactions, and metal oxides are then formed via dehydration [26].

Although the fluid used in this study was hexane and not water, some of the corrosion reactions that occur in water may have occurred owing to the presence of dissolved oxygen and moisture in hexane.

**Figure 7:**
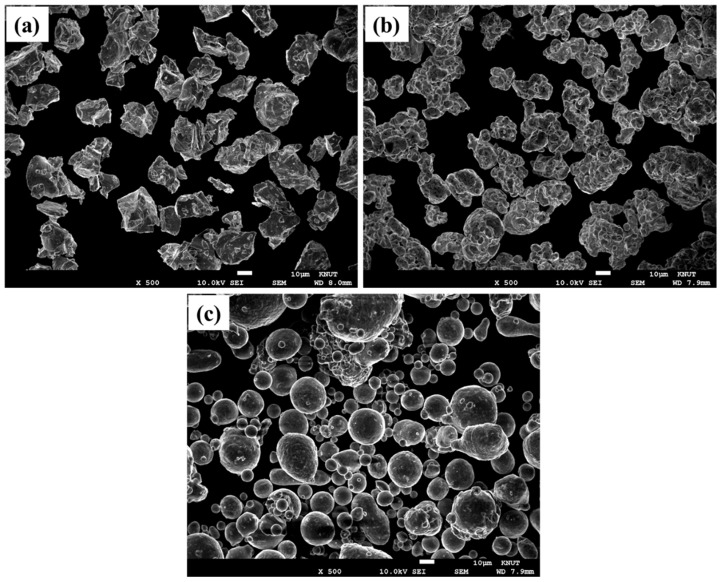
SEM micrographs of the metal powders before the purification of hexane: (**a**) Ti, (**b**) Cu, and (**c**) Al.

Another possible reason for the low-temperature oxidation, though insignificant, may have been the acceleration of corrosion because of the buoyant pressure experienced by the metal powder in the fluid [27]. Next, we examined different oxidation behaviors by material by investigating the factors that may have affected them, such as the specific surface area and intrinsic surface energy of each material and the shape of the metal particles [28,29]. The specific surface areas of Ti, Cu and Al powders were found to be 0.1548, 0.055, and 0.1221 m^2^/g, respectively [30]. Thus, the specific surface area of the Ti powder was slightly higher than those of the Cu and Al powders. The surface energies of Ti, Cu, and Al have been reported to be 2.034, 1.793, and 0.864 J/m^2^ [26], respectively, with Ti exhibiting the highest surface energy. Therefore, the relatively high specific surface area and surface energy of Ti powder are believed to have contributed to its high oxidation reaction rate [31,32]. Moreover, the differences in the surface areas may have been related to particle shape as well [33]. Hence, it was necessary to examine the initial particles of metal powders used in the purification experiment. Figure 7 shows SEM images of the microstructures of the metal powders before the purification of hexane. Ti, Cu, and Al powders were observed to have average particle sizes of approximately 30–40 μm. It can be observed that the Al powders are nearly spherical in shape, and the Ti powder exhibits completely irregular morphology. By contrast, the Cu powder exhibits neither completely spherical nor irregular morphology. From Figure 7a it is clear that the particle shape of the Ti powder, which underwent the hydrogenation–dehydrogenation (HDH) and pulverization processes, was relatively irregular than those of the Cu and Al powders manufactured via atomization processes. The irregular particle shape may have resulted in higher surface energy than the spherical shape, which is qualitatively consistent with the previously described surface energy results [34,35]. By contrast, at relatively high temperatures of 308 and 323 K, the weight change of Cu and Al powder was larger than that of Ti; this will be examined in future research.

Table 2 presents the results of the carburization of tantalum powder using purified and unpurified hexane. The carbon content of TaC was consistent with its theoretical value of 6.22 wt.% [36], regardless of whether Ti powder was used. However, in the case of carburization using unpurified hexane, the oxygen content of the prepared TaC was approximately six times higher than that of the TaC obtained by purified hexane.

**Figure 8:**
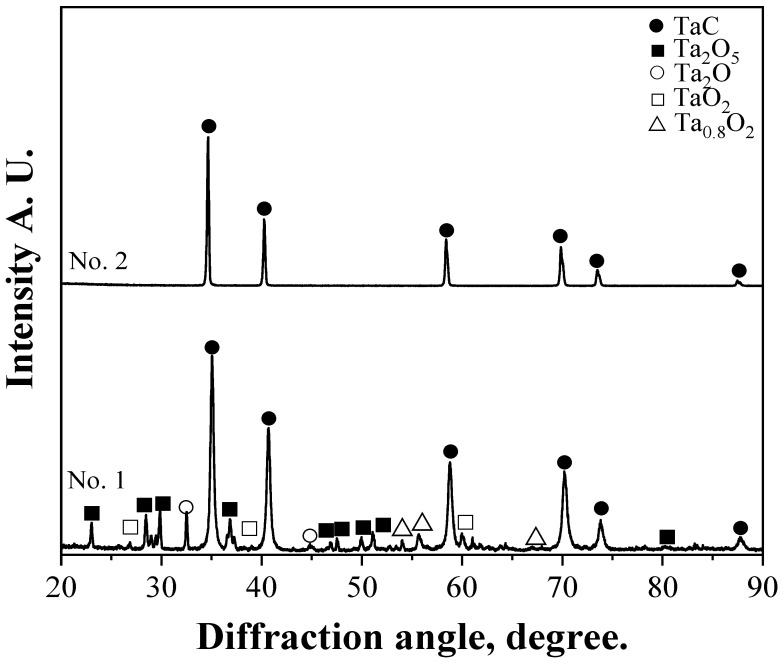
X-ray diffraction analysis of Ta samples carburized using purified hexane (**No. 2**) and unpurified hexane (**No. 1**) over 2 h.

Figure 8 shows the XRD results of the TaC powder referred to in Table 2. The results revealed the well-formed TaC phase in the carburization using hexane with Ti powder. Meanwhile, in the carburization using hexane without Ti powder, many oxide phases, such as Ta_2_O, TaO_2_, Ta_0.8_O_2_, and Ta_2_O_5_ were present, indicating that oxidation occurred during carburization. This is qualitatively consistent with the high-oxygen-content results of the carburization experiment using unpurified hexane (Table 2). By contrast, the oxide products of Ta were completely absent in the sample carburized using purified hexane (No. 2). This demonstrates the efficiency of the titanium-purified hexane as a carburizing medium.

## 4. Conclusions

Tantalum carbide powder was prepared via the carburization of tantalum metal powder produced a previous study, using purified hexane gas. The changes in the weights of the Ti, Cu, and Al powders used for hexane purification were +0.083, +0.121, and +0.065 g, respectively, at 308 K, and +0.086, +0.167, and +0.086 g, respectively, at 323 K, which were attributed to the corrosion of the metals. The differences in the weight changes of the powders were attributed to the differences in their specific surface areas, surface energies, and particle shapes. Compared with other powders (Al and Cu), the lower activation energy of Ti results in a higher oxidation rate in hexane at room temperature. This indicates that Ti is more effective at removing oxygen-containing impurities from hexane at room temperature than copper and aluminum. In the carburizing reaction, the carbon content of the tantalum product was almost identical to the theoretical value, regardless of whether hexane had been purified, indicating successful carburization. Moreover, the oxygen content in the tantalum carburized using purified hexane was approximately six times lower than that of tantalum carburized using unpurified hexane, indicating that hexane purified using Ti powder yielded high-purity tantalum carbide, without producing undesirable oxidation products. Through this, we produced high-purity tantalum carbide faster and more efficiently than the traditional carburizing approach using a technology that used less energy and more safety. Thereafter, research on manufacturing tantalum carbide of higher purity is planned by further performing a process of removing impurities of hydrocarbon used before the carbonization process.

## Figures and Tables

**Table 1:** Oxidation rates of the metal powders used for the purification of hexane at various stirring temperatures.

Material	T, K	Δm, g	1/T, 1/K	Δm/3 h = k
Titanium	293	0.081	3.413 × 10^−3^	0.0269
	308	0.083	3.247 × 10^−3^	0.0277
	323	0.086	3.096 × 10^−3^	0.0285
Copper	293	0.005	3.413 × 10^−3^	0.0018
	308	0.121	3.247 × 10^−3^	0.0404
	323	0.167	3.096 × 10^−3^	0.0557
Aluminum	293	0.000	3.413 × 10^−3^	0.0001
	308	0.065	3.247 × 10^−3^	0.0217
	323	0.086	3.096 × 10^−3^	0.0285

**Table 2:** Carburization of reduced tantalum powder using hexane purified using Ti powder (O) and unpurified hexane (X) at 1273 K over 2 h.

No.	Purification by Ti Powder	T, K	Time, h	C, wt.%	O, wt.%
1	X	1273	2	6.22	7.66
2	O	1273	2	6.23	1.39

## Data Availability

Not applicable.
